# Single-Cell RNA Sequencing Shows Exercise Protects *db/db* Mouse Pancreatic Injuries by Regulating Endothelial Cell Homeostasis

**DOI:** 10.1155/jdr/9676094

**Published:** 2025-09-22

**Authors:** Wenya Weng, Manjuan Li, Zixuan Wang, Mengmeng Peng, Guoyong Fan, Shicui Jiang, Chi Zhang, Xuemian Lu

**Affiliations:** Department of Endocrinology, The Third Affiliated Hospital of Wenzhou Medical University, Wenzhou City, Zhejiang Province, China

**Keywords:** capillary endothelial cells, interval aerobic training, nonpharmacological treatment, Type 2 diabetes, vascular development

## Abstract

The prevalence and mortality rates of Type 2 diabetes mellitus (T2DM) continue to increase, imposing a significant burden on individuals and nations worldwide. The pancreas plays an important role in T2DM development, while exercise is a crucial nonpharmacological treatment. Although the pancreas comprises various cell types, current studies on the effects of exercise on diabetes have mainly focused on the beta cells. In this study, we aimed to enhance our understanding of the effects of exercise on other pancreatic cell types. This was aimed at facilitating the comprehensive analysis of the processes and principles by which exercise protects and enhances pancreatic function. Six-week-old male *db/db* mice were trained on a treadmill at a speed of 9 m/min for 10 weeks (50 min/day, 5 days/week). Single-cell RNA sequencing (scRNA-seq) was performed to analyze cell types in the pancreas. The results showed that exercise improved the body weight and pancreas profile of *db/db* mice. The scRNA-seq demonstrated that the pancreas was composed of 12 cell types, with obvious changes in endothelial cells (ECs) among all groups. Further subtype analysis suggested that ECs could be annotated into five subtypes, with capillary ECs and unknown EC 1 presenting remarkable differences among the groups. Additionally, Gene Ontology (GO) enrichment analysis showed the roles of regulation of EC proliferation and response to injury for capillary ECs and unknown EC 1, respectively. The two EC subtypes may be involved in the protective effect of exercise against pancreatic injury in *db/db* mice.

## 1. Introduction

The International Diabetes Federation (IDF) Diabetes Atlas 2021 shows that over 500 million people have diabetes globally. This number has been inferred to reach 643 and 783 million by 2030 and 2045, respectively [1]. Type 2 diabetes mellitus, or T2DM, is the predominant form of the disease, comprising over 90% of all diabetes cases worldwide, while its high global prevalence is increasing across all regions [1]. Although the precise causes of T2DM remain elusive, it is well established that the condition is closely associated with several risk factors, including obesity, advancing age, certain ethnic backgrounds, and a family history of diabetes. The foundation of T2DM management lies in adopting a lifestyle that supports good health, which encompasses a nutritious diet, regular exercise, abstaining from smoking, and maintaining a healthy body weight [1].

Throughout history, exercise and similar activities have traditionally been viewed as leisure pursuits in earlier civilizations. They have only gradually evolved into a form of preventative and therapeutic intervention in more recent times [2]. Exercise can be categorized based on the type of energy it uses, falling into categories such as aerobic, anaerobic, or a blend of the two. It primarily focuses on enhancing muscular and cardiovascular fitness. Moreover, regular exercise, especially aerobic activities, has been found to support the immune system and may help counteract the effects of reduced adaptive responses and chronic inflammation.

Engaging in regular, moderate exercise is associated with a lower risk of infections compared to a sedentary lifestyle [3], and exercise is a crucial nonpharmacological treatment for diabetes. Regular exercise not only reduces blood pressure and circulating lipid levels but also decreases the risk of cardiovascular issues. Furthermore, it enhances insulin sensitivity and promotes glucose uptake by skeletal muscle cells, which can help in managing body weight, particularly beneficial for individuals with Type 1 diabetes mellitus (T1DM) and T2DM [4, 5]. Compelling evidence has shown that interval aerobic training may be more beneficial than continuous aerobic training in preventing the onset or development of T2DM [6, 7].

The pancreas, which is an exocrine and endocrine gland, is the primary organ involved in nutrient metabolism and is known to play an important role in T2DM. It functions as a “control hub” for several major glucoregulatory hormones and plays a significant role in the production and secretion of numerous enzymes that aid in nutrient digestion and absorption [8].

In mammals, the pancreas originates from two mutually independent buds, the ventral and dorsal pancreatic buds. These buds are home to undifferentiated progenitor cells that have the remarkable ability to transform into all types of pancreatic cells, such as those found in the acinar, ductal, and endocrine lineages [9, 10]. Pancreatic stellate cells are versatile with exocrine functions in the pancreatic region [11]. Stimulation by pancreatic injury, inflammation, or toxic substances activates cells such as macrophages, platelets, acinar cells, ductal cells, and endothelial cells (ECs). This induces secretion of cytokines that promote pancreatic stellate cell activation through paracrine signaling, ultimately leading to pancreatic fibrosis [12–16].

The pancreatic endocrine function is derived from hormone-secreting epithelial clusters called islets of Langerhans that are mainly composed of five cell types: *α*, *β*, *δ*, *ε*, and PP cells [9]. The *α* cells are responsible for producing glucagon, a hormone that raises blood glucose levels. Conversely, the *β* cells generate insulin, which lowers blood glucose levels. This delicate balance ensures that blood glucose concentrations remain within a narrow range, supplying energy to the body's active tissues [17–19]. The *δ* cells produce somatostatin, which inhibits both glucagon and insulin release [20]. PP cells produce pancreatic polypeptides that regulate the exocrine and endocrine secretory activities of the pancreas [21]. Collectively, these hormones regulate glucose homeostasis in vertebrates [19].

Islets contain numerous microvessels that morphologically form structures similar to kidney glomeruli and are primarily composed of ECs, which are critical to maintaining normal islet function. ECs meet the nutritional needs of islets, enabling them to rapidly sense changes in the metabolic environment of the body and quickly secrete various hormones to regulate the metabolic balance. They also interact with endocrine cells, participating in physiological and pathological processes, such as the development of pancreatic endocrine function, induction of insulin gene expression, inflammatory response damage to the islets, and revascularization of transplanted islets [22]. Islet ECs participate in supplying essential nutrients to the islet endocrine cells and play a significant role in the development, proliferation, and functional regulation of islet *β* cells [23].

Currently, studies on the effects of exercise on diabetes have mainly focused on its impact on *β* cells, with the aim of improving diabetes [24–26]. Studies on human islets and animal models suggested that physical training may have a direct beneficial effect on pancreatic *β* cells through circulating mediators [24–26]. For instance, Paula et al. [25] suggested that exercise serves as a protective measure for both human and rodent *β* cells against endoplasmic reticulum (ER) stress and apoptosis, which are heightened under conditions of diabetes. However, this does not account for the existence of the other various cell types, as described previously.

Although one cause of T2DM in mice is the destruction of *β* cells, studies suggest that other cells in the pancreas can affect the proliferation of *β* cells and that hyperglycemia can lead to pathological changes in multiple pancreatic cell types [27, 28]. For instance, recent studies in animal models support the role of activated pancreatic stellate cells in the development of islet fibrosis and *β* cell dysfunction during T2DM [29, 30]. Furthermore, single-cell studies have revealed that pancreatic ECs exhibit unique organ-specific transcriptional signatures, including PLPP1 and CDC42, that regulate islet–vascular crosstalk [31]. Their functional heterogeneity directly governs islet blood supply and *β* cell survival, as evidenced by PPAR*δ*-mediated metabolic coupling between ECs and *β* cells in diabetic models [32, 33]. It is reported that diabetic ECs display dysfunctions such as eNOS uncoupling and reduced nitric oxide (NO) bioavailability [34]. This leads to the generation of reactive oxygen species (ROS), which further intensifies the oxidative damage to *β* cells [35]. Alarmingly, this detrimental process forms a vicious cycle with the inherently weak antioxidant capacity of *β* cells [36].

The responses of other pancreatic cells to dynamic physical exercise under diabetic conditions are less well understood than those of *β* cells. Recent single-cell evidence identifies organ-specific EC responses, with hippocampal ECs showing Notch pathway activation postexercise [37], providing a theoretical foundation for investigating exercise-induced pancreatic EC remodeling. Evidence also shows that exercise improves endothelial function by increasing NO bioavailability and reducing oxidative stress [38]. Therefore, understanding the effects of exercise on other cell types would facilitate the comprehensive elucidation of the processes and principles by which exercise protects and enhances pancreatic function.

Single-cell RNA sequencing, or scRNA-seq, is a cutting-edge technique that involves sequencing and genome analysis at the individual cell level. This technique demonstrates powerful capabilities in exploring cell heterogeneity, discovering new cell types, and revealing novel molecular regulatory mechanisms [39].

We have recently identified two subtypes of ECs that are potentially involved in the protective effects of exercise against pancreatic injury in *db/db* mice. Therefore, our study was aimed at utilizing scRNA-seq to further examine changes in pancreatic cell composition, status, and characteristics to identify possible pancreatic cells associated with exercise-related protection against pancreatic impairment. In addition, we sought to provide new insights into the restoration of pancreatic damage through exercise.

## 2. Materials and Methods

### 2.1. Animals

The 6-week-old spontaneously diabetic *db/db* mice, a model used to study noninsulin-dependent T2DM, were purchased from Changzhou Cavens Laboratory Animal Co. Ltd. The mice were randomly separated into the following three groups: control (*db/m*, *n* = 8), untreated diabetic (*db/db*, *n* = 10), and exercise diabetic group (*db/db*-Ex, *n* = 11). All animals were housed in special pathogen-free cages and allowed free access to food and water on a 12-h day/night cycle. They were handled in accordance with the approved protocols and guidelines for the care and use of laboratory animals of the Third Affiliated Hospital of Wenzhou Medical University Animal Policy and Welfare Committee (Institutional Review Board [IRB] Number SYXK2020-0014, Zhejiang, China).

### 2.2. Treadmill Exercise

Six-week-old male *db/db* mice were trained on a treadmill for acclimation at a 10° uphill incline for 5 days. On Day 1, the running speed was 8 m/min for 5 min, which was then extended to 10 min on Days 2–5 at the same speed.

#### 2.2.1. Formal Training

At 7 weeks old, male *db/db* mice were formally trained on the treadmill at a speed of 9 m/min for a duration of 50 min/day for 5 days/week, consecutively for 10 weeks.

### 2.3. Blood Glucose and Body Weight Monitoring and Sample Collection

Blood glucose levels and body weight of the mice were recorded weekly. At the 16-week-old final checkpoint, a blood glucose meter was used to measure fasting blood glucose (FPG). The mice were then euthanized, and their body weight was measured, followed by collection of pancreatic tissue and blood samples. The pancreas was stored in liquid nitrogen until further analysis.

### 2.4. Histopathological Examination

The pancreatic tissue samples were fixed in formalin in 10% phosphate-buffered saline for 24 h, embedded in paraffin, and cut into 5-*μ*m-thick sections for pathological analysis after dehydrating in a gradient series of alcohol solutions (75%, 85%, 95%, and 100%). For general morphological detection, hematoxylin and eosin (H&E) staining was used, and collagen accumulation was distinguished using Masson's trichrome staining.

### 2.5. Terminal Deoxynucleotidyl Transferase (TdT)–Mediated Deoxyuridine Triphosphate (dUTP) Nick End Labeling (TUNEL) Assay

The tissue slices underwent a two-step incubation process: first, they were soaked in a 30% hydrogen peroxide solution for 30 min at room temperature (25°C). After that, they were exposed to proteinase K for 40 min at a higher temperature of 37°C. Subsequently, the slices were incubated in a reaction mixture containing TdT and digoxigenin-labeled dUTP for 60 min at 37°C. For the negative control, the reaction mixture was used without TdT. The stained sections were then visualized using 3,3⁣′-diaminobenzidine (DAB) as a chromogen.

### 2.6. scRNA-seq Analysis and Bioinformatics Analysis Workflow

The 10x Genomics platform, which uses microfluidic technology to sort individual cells, was used in this study.

#### 2.6.1. Quality Control (QC) During Data Processing

Before proceeding with the main analysis, several QC steps were implemented to ensure the reliability of the data and to filter out low-quality cells.

1. Cell Ranger processing: The official analysis software Cell Ranger (10x Genomics, https://support.10xgenomics.com/single-cell-gene-expression/software/overview/welcome) was used to process the raw data. First, reads with low base-call quality scores (Phred score < 20) were removed. Reads that did not map to the reference genome (*Mus musculus* genome, Ensembl v105) were discarded.
2. Seurat analysis: Seurat (https://satijalab.org/seurat/, v4.1.0.) was used for further cell filtering, standardization, cell subtype classification, analysis of differentially expressed genes (DEGs) within each subtype, and selection of marker genes. For cell identification, cells with fewer than 200 detected genes were considered low quality and were filtered out. Additionally, cells with a mitochondrial gene content greater than 25% were also removed, as a high mitochondrial gene content may indicate cell stress or damage. And DoubletFinder (v2.0.3) was used to remove doublet cells. After these filtering steps, techniques such as alignment, quantification, and identification of recovered cells were performed, ultimately resulting in a gene expression matrix for each cell type. After that, the data was normalized using the LogNormalize method in Seurat, with a scale factor of 10,000.

For cell subtype classification, the FindVariableFeatures function in Seurat was used to identify highly variable genes (HVGs). The top 2000 HVGs were selected based on their dispersion and mean expression levels. These HVGs were then used for principal component analysis (PCA). The number of significant principal components (PCs) was determined using the Elbow method and selected as 20. Based on the significant PCs, cells were clustered using the FindClusters function with a resolution of 0.8.

DEGs within each subtype were identified using the FindMarkers function in Seurat, with a log2 fold change threshold of 0.26 and a *p* value threshold of 0.01. Marker genes for each cell subtype were selected based on their specificity and high expression levels within the subtype.

### 2.7. Statistical Analysis

The results of repeated experiments are presented as averages ± standard deviations (SD). To compare the different groups, we used an analysis of variance (ANOVA). We considered statistical significance at *p* < 0.05.

## 3. Results

### 3.1. Positive Effects of Exercise on Body Weight and Pancreas Profile in *db/db* Mice

**Figure 1:**
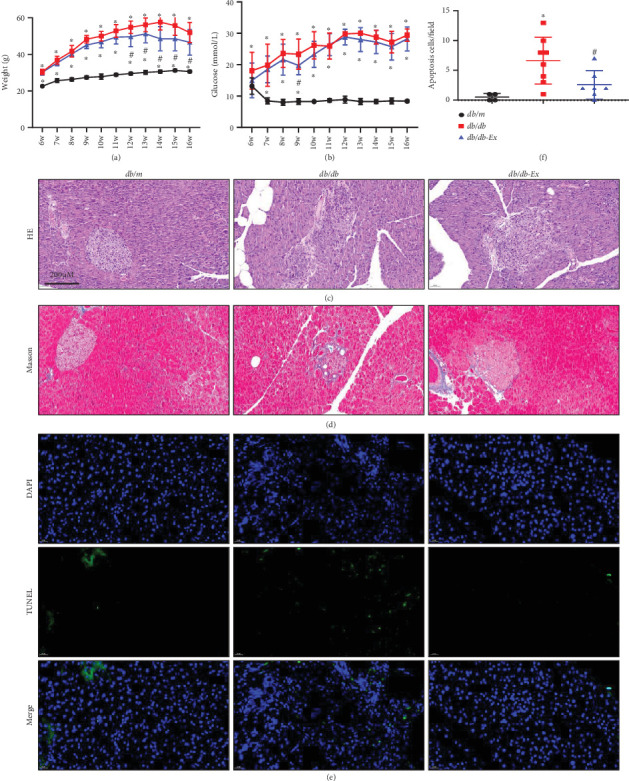
The protective effects of exercise on body weight and pancreas profile in *db/db* mice. Weekly body weight before sacrifice (a) and weekly blood glucose before sacrifice (b). Pancreas pathology was examined with hematoxylin and eosin (H&E) staining (c) and Masson staining (d). Apoptosis was examined by terminal deoxynucleotidyl transferase–mediated dUTP nick end labeling (TUNEL) staining (green), nuclei staining with 4⁣′,6-diamidino-2-phenylindole (DAPI) (blue), and merge (E&F) (×400). ⁣^∗^*p* ≤ 0.05 (vs. *db/m*), ^#^*p* ≤ 0.05 (vs. *db/db*).

Our results demonstrated that at 6 weeks old, the body weight of the *db/db* mice was greater than that of the db/m mice, whereas there was no difference between the *db/db* and *db/db*-Ex groups. After 5 weeks of training, the body weight of *db/db*-Ex mice decreased significantly compared to that of the untrained *db/db* mice. This significant weight reduction continued until completion of the 10-week exercise program (Figure 1a). However, 10 weeks of training did not obviously change the blood glucose levels of the *db/db*-Ex mice compared with those of *db/db* mice, although the *db/db*-Ex group showed a lower level than that of the *db/db* group at 9 weeks old (Figure 1b). Furthermore, the pathological analysis results showed that exercise rescued the diabetes-induced phenomenon of islet cell cavitation degeneration, unclear islet boundaries (Figure 1c), islet fibrosis, and inward displacement of the exocrine glands (Figure 1d). In addition, the TUNEL results showed that diabetes exacerbated apoptosis of the pancreas, whereas exercise remarkably alleviated the apoptosis (Figure 1e,f).

### 3.2. scRNA-seq Identified Pancreatic Cell Types in *db/db* Mice

**Figure 2:**
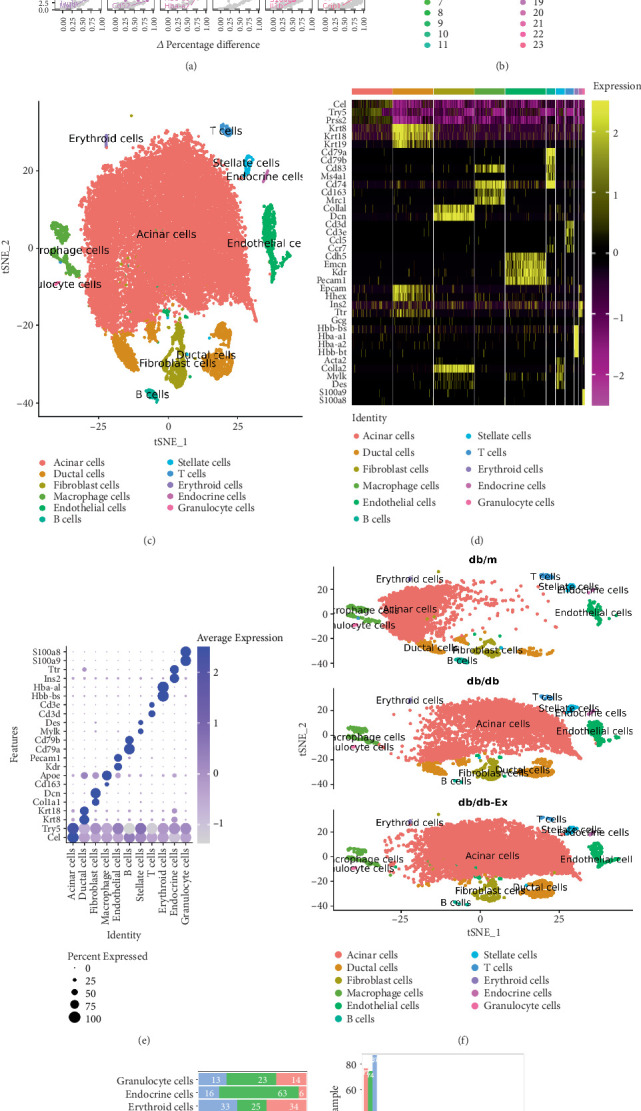
scRNA-seq reveals pancreatic cell types in *db/db* mice. The main major genes in each cluster (a). UMAP projection of pancreatic cells, colored by assigned cluster (b). t-SNE plot of pancreatic single-cell (sc) RNA-seq profiles, colored by cluster assignment and annotated post hoc (c). Heatmap of cell type–enriched genes. Each column represents a single cell and each row represents one signature gene. The colors ranging from purple to yellow indicate low to high relative gene expression levels (d). Dot plots of representative two specific genes in each cell type (e). t-SNE plot of pancreatic single-cell (sc) RNA-seq profiles of three groups, colored by cluster assignment and annotated post hoc (f). Normalized frequencies of populations (g) and fraction of cells in each population relative to the total number of pancreatic cells (h).

In our study, we implemented a comprehensive QC pipeline to filter out low-quality cells, which included the assessment of mitochondrial gene content. Cells with fewer than 200 detected genes were considered low quality and were filtered out. Additionally, cells with a mitochondrial gene content greater than 25% were also removed, as a high mitochondrial gene content may indicate cell stress or damage. After these filtering steps, we performed alignment, quantification, and identification of recovered cells, ultimately generating a gene expression matrix for each cell type. The data was then normalized using the LogNormalize method in Seurat with a scale factor of 10,000 (Supporting Information 1: Material S1). PCA of the 2000 most variable genes revealed the key genes in each cluster (Figure 2a). Additionally, Figure 2b illustrates that all cells were grouped into 23 clusters based on their marker genes. These clusters were then classified into 12 distinct cell types (Figure 2c) according to the Mouse Cell Atlas database (https://bis.zju.edu.cn/MCA/gallery.html) and reported literature, with one unknown cell type included [40, 41]. The top 10 DEGs for each cell type in the pancreas are presented in Supporting Information 2: Material S2.

The specific genes used for the annotation of each cell type are shown in Figure 2d. For example, pancreatic lipase–related protein 1 (*Pnliprp1*), carboxyl ester lipase (*Cel*), trypsin 5 (*Try5*), and serine protease 2 (*Prss2*) were particularly expressed in acinar cells, whereas keratin 8 (*Krt8*), *Krt18*, and *Krt19* were expressed in ductal cells and CD79A antigen (immunoglobulin-associated alpha) (*Cd79a*), *Cd79b*, *Cd83*, and membrane-spanning 4-domains, subfamily A, member 1 (*Ms4a1*) were expressed in B cells.

Furthermore, *Cd3d*, *Cd3e*, C-C motif chemokine ligand 5 (*Ccl5*), and C-C motif chemokine receptor 7 (*Ccr7*) were expressed in T cells; *Cd74*, *Cd163*, and mannose receptor, C type 1 (*Mrc1*) were expressed in macrophages; collagen, type I, alpha 1 (*Col1a1*), and decorin (*Dcn*) were expressed in fibroblast cells; and cadherin 5 (*Cdh5*), endomucin (*Emcn*), kinase insert domain protein receptor (*Kdr*), and platelet/endothelial cell adhesion molecule 1 (*Pecam1*) were expressed in ECs.

In addition, epithelial cell adhesion molecule (*Epcam*) was expressed in epithelial cells; hematopoietically expressed homeobox (*Hhex*), insulin II (*Ins2*), transthyretin (*Ttr*), and glucagon (*Gcg*) were expressed in endocrine cells; and hemoglobin, beta adult s chain (*Hbb-bs*), hemoglobin alpha, adult chain 1 (*Hba-a1*), *Hba-a2*, and hemoglobin, beta adult t chain (*Hbb-bt*) were expressed in erythroid cells. Actin alpha 2, smooth muscle, aorta (*Acta2*), *Col1a2*, myosin, light polypeptide kinase (*Mylk*), and desmin (*Des*) were expressed in stellate cells; and S100 calcium binding protein A9 (calgranulin B, *S100a9*) and *S100a8* were expressed in granulocyte cells.

Figure 2e shows two representative genes in each cell type as a dotted plot. The numbers and percentages of different cell types varied among the three groups (Figures 2f, 2g, and 2h), indicating that the sample data of the three groups were quite different. More specifically, the percentage of ECs in the *db/db* group (1.76%) was significantly higher than that in the *db/m* group (6.09%), which was reduced to 2.85% by exercise. The same trend was observed in the fibroblasts, where the proportion increased from 3.55% in the *db/m* group to 5.09% in the *db/db* group and sharply decreased to 2.51% in the *db/db*-Ex group.

However, the percentage of ECs in the *db/db*-Ex group was even lower than that in the *db/m* group, which was inexplicable. Although the trends were similar in ductal, stellate, endocrine, and granulocyte cells, these differences were not significant. Furthermore, the trend in acinar cells was the opposite of that in the above cells, with no obvious reduction in the *db/db* group compared to the *db/m* group (Figure 2h and Table 1).

### 3.3. scRNA-seq Annotated Five EC Subtypes in Mouse Pancreas

**Figure 3:**
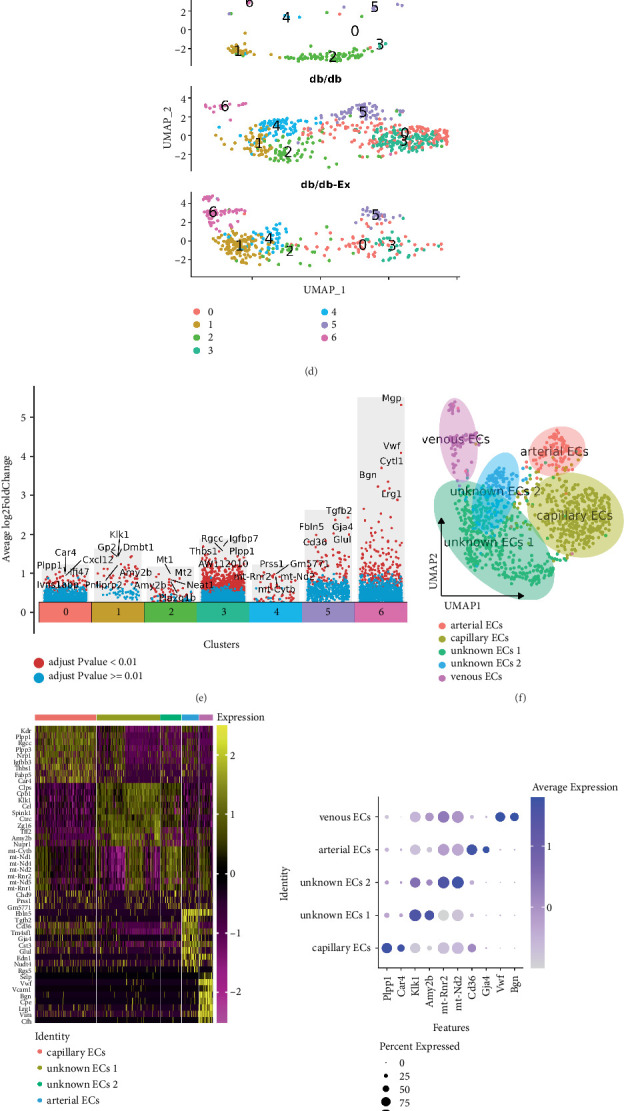
scRNA-seq annotated five endothelial cell subtypes in mouse pancreas. We analyzed endothelial cells (a). The top 10 differentially expressed genes (DEGs) in endothelial cells (b). UMAP projection of pancreatic endothelial cells, colored by assigned cluster (c). UMAP projection of pancreatic cells of three groups, colored by assigned cluster (d). The major genes in each cluster (e). UMAP projection of pancreatic endothelial cells, colored by cluster assignment and annotated post hoc (f). Heatmap of cell type–enriched genes. Each column represents a single cell and each row represents one signature gene. The colors ranging from purple to yellow indicate low to high relative gene expression levels (g). Dot plots of representative two specific genes in each cell type (h). UMAP projection of pancreatic endothelial cells of three groups, colored by cluster assignment and annotated post hoc (i). Normalized frequencies of populations (j) and fraction of cells in each population relative to the total number of pancreatic cells (k).

Our analysis of the ECs identified the following as the top 10 DEGs: fatty acid binding protein 4, adipocyte (*Fabp4)*, Fms-related receptor tyrosine kinase 1 (*Flit1*), lymphocyte antigen 6 family member C1 (*Ly6c1*), plasmalemma vesicle-associated protein (*Plvap*), insulin-like growth factor binding protein 7 (*Igfbp7*), *Kdr*, protein tyrosine phosphatase receptor type B (*Ptprb*), GPI-anchored HDL-binding protein 1 (*Gpihbp1*), insulin-like growth factor binding protein 3 (*Igfbp3*), and *Ly6a* (Figure 3b). Further analysis revealed that the pancreatic ECs consisted of seven clusters of subtypes, 0–6 (Figure 3c).

Figure 3d shows that the number of EC subtypes varied dramatically among the three groups. The major genes in each cluster are shown in Figure 3e, and the cells were divided into seven clusters based on different marker genes. The seven clusters were then divided into five cell subtypes: capillary, arterial, venous, unknown 1, and unknown 2 (Figure 3f). The two clusters expressed multiple EC marker genes but could not be assigned to a well-described EC subtype (unknown 1 and unknown 2).  Figure 3g shows the specific genes used to annotate each cell subtype. The top 10 DEGs of the subcell types of pancreatic ECs are displayed in Supporting Information 3: Material S3.

For example, *Kdr*, phospholipid phosphatase 1 (*Plpp1*), regulator of cell cycle (*Rgcc*), *Plpp3*, neuropilin 1 (*Nrp1*), *Igfbp3*, thrombospondin 1 (*Thbs1*), fatty acid binding protein 5, epidermal (*Fabp5*), and carbonic anhydrase 4 (*Car4*) were specifically expressed in capillary ECs, whereas fibulin 5 (*Fbln5*), transforming growth factor, beta 2 (*Tgfb2*), *Cd36*, transmembrane 4 superfamily member 1 (*Tm4sf1*), gap junction protein, alpha 4 (*Gja4*), cystatin C (*Cst3*), glutamate synthase 1 (*Glu1*), endothelin 1 (*Edn1*), nudix hydrolase 4 (*Nudt4*), and regulator of G-protein signaling 5 (*Rgs5*) were expressed in arterial ECs.

Selectin, platelet (*Selp*), Von Willebrand factor (*Vwf*), vascular cell adhesion molecule 1 (*Vcam1*), biglycan (*Bgn*), carboxypeptidase E (*Cpe*), leucine-rich alpha-2-glycoprotein 1 (*Lrg1*), vimentin (*Vim*), and complement component factor h (*Cfh*) were expressed in venous ECs [42–46].

Colipase, pancreatic (*Clps*), carboxypeptidase B1 (*Cpb1*), kallikrein 1 (*Klk1*), *Cel*, serine peptidase inhibitor, Kazal type 1 (*Spink1*), chymotrypsin C (*Ctrc*), zymogen granule protein 16 (*Zg16*), trefoil factor 2 (spasmolytic protein 1, *Tff2*), amylase 2b (*Amy2b*), and nuclear protein transcription regulator 1 *(Nupr1*) were expressed in the unknown EC 1. Furthermore, cytochrome b, mitochondrial (*mt-Cytb*), NADH dehydrogenase 1, mitochondrial (*mt-Nd1*), *mt-Nd4*, *mt-Nd2*, 16S ribosomal RNA (*mt-Rnr2*), *mt-Nd5*, *mt-Rnr1*, chromodomain helicase DNA binding protein 9 (*Chd9*), *Prss1*, and serine protease 1 (trypsin 1) like (*Prss1l*, *Gm5771*) were expressed in the unknown 2 ECs.

Figure 3h shows two representative genes in each cell subtype as a dotted plot. The cell numbers (Figure 3i,j) of the different cell subtypes and the percentages of all cell subtypes (Figure 3k and Table 2) varied greatly among the three groups. Specifically, the percentage of capillary ECs in the *db/db* group significantly exceeded that in the *db/m* group, from 3.5% to 49.49%, whereas there was a sharp decrease to 24.1% during exercise. A similar trend was observed with the arterial ECs, which increased from 5.59% in the *db/m* group to 11.77% in the *db/db* group, whereas they decreased to 7.69% in the *db/db*-Ex group.

However, the number of arterial ECs was too small to be considered reliable. Although the trend was similar for the unknown EC 2 subtype, the deviations were not significant. Furthermore, the trend with the unknown EC 1 subtype was opposite to that observed with the capillary cell subtype. Moreover, exercise obviously reversed the effect by reducing the effect of diabetes (Figure 3k and Table 2).

### 3.4. Heterogeneity of Pancreatic EC Subtypes Among Different Groups

**Figure 4:**
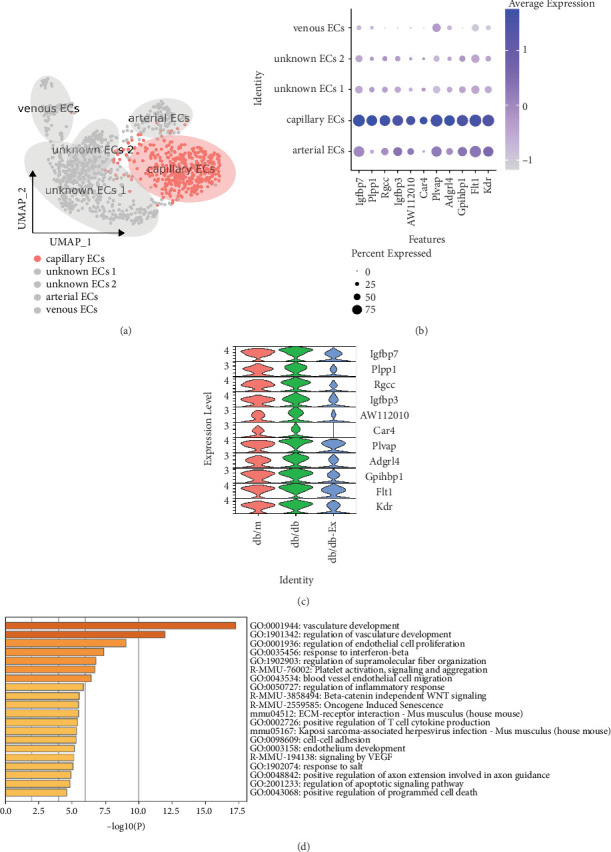
Heterogeneity of pancreatic endothelial cell subtypes among different groups. We analyzed the differences in capillary ECs among three groups (a). The top 10 most commonly expressed genes in capillary ECs (b). Differences of the top 10 most commonly expressed genes in capillary ECs among three groups (c). Gene Ontology enrichment analysis (GO enrichment) of marker genes (log2FC > 0.9) in capillary ECs (d).

Next, we analyzed the differences in capillary ECs among the three groups (Figure 4a). First, we examined the following 10 most commonly expressed genes in the capillary ECs: *Igfbp7*, *Plpp1*, *Rgcc*, I*gfbp3*, AW112010, *Car4*, *Plvap*, adhesion G protein-coupled receptor L4 (*Adgrl4*), GPI-anchored HDL-binding protein 1 (*Gpihbp1*), and *Kdr* (Figure 4b). The results showed that the expression levels of all 10 capillary EC-specific genes were higher in the *db/db* group than they were in the *db/m* group, whereas exercise drastically downregulated the expression levels of these genes in *db/db* mice (Figure 4c).

**Figure 5:**
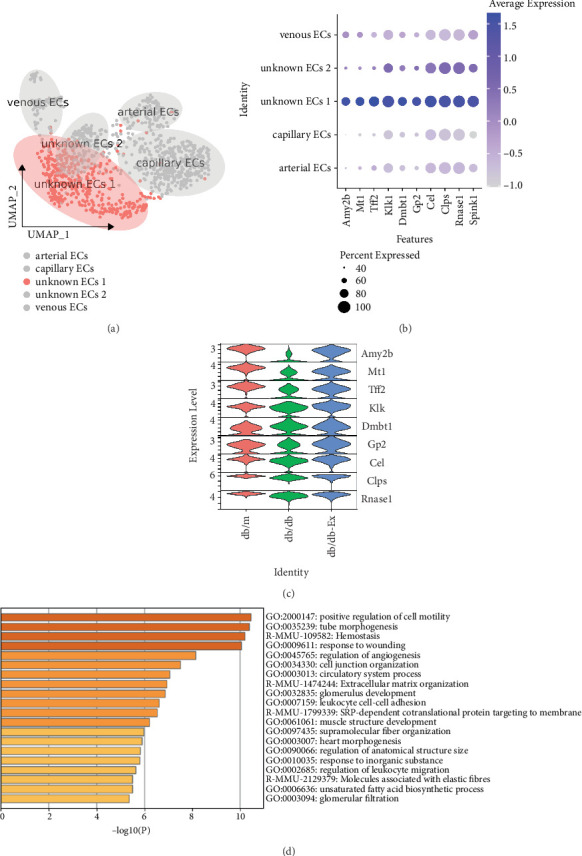
Heterogeneity of pancreatic endothelial cell subtypes among different groups. We analyzed the differences in unknown EC 1 among three groups (a). The top 10 most commonly expressed genes in unknown EC 1 (b). Differences of the top 10 most commonly expressed genes in unknown EC 1 among three groups (c). Gene Ontology enrichment analysis (GO enrichment) of marker genes (log2FC > 0.9) in unknown EC 1 (d).

Gene Ontology (GO) enrichment analysis revealed typical signaling pathways involving marker genes (log2FC > 0.9, Figure 4d), and we used the same method to analyze the top 10 genes specifically expressed in the unknown EC 1 (Figure 5a). The results showed that *Amy2b*, metallothionein 1 (*Mt1*), *Tff2*, *Klk1*, deleted in malignant brain tumors 1 (*Dmbt1*), glycoprotein 2 zymogen granule membrane (*Gp2*), *Cel*, *Clps*, ribonuclease, RNase A family, 1 (pancreatic, *Rnase1*), and *Spink1* were particularly abundant in unknown EC 1 (Figure 5b). In contrast, almost all these genes showed the opposite trend to that shown in capillary ECs in the three groups (Figure 5c).

Similarly, GO enrichment analysis of the signaling pathways related to the top marker genes in unknown EC 1 (log2FC > 0.9) revealed the following: positive regulation of cell motility, tubal morphogenesis, homeostasis, response to injury, regulation of angiogenesis, cell junction organization, circulatory system process, extracellular matrix organization, leukocyte cell–cell adhesion, and SRP-dependent cotranslational protein targeting the membrane (Figure 5d).

## 4. Discussion

In this study, we investigated the effects of exercise on various pancreatic cell types other than the widely studied *β* cells, in a model of 6-week-old male *db/db* mice that received daily exercise training for 5 days/week for 10 weeks. The results showed that exercise significantly reduced the body weight of the T2DM mice and repaired their pancreatic tissue structure. Moreover, the exercise-induced reduction in apoptotic cells further supported the positive effects of exercise in alleviating pancreatic damage in the diabetic mice.

However, we found that exercise did not significantly lower blood glucose levels in *db/db* mice, which contradicted expectations. The disease progresses rapidly and *β* cell exhaustion is severe in this mouse model [47]. Insulin resistance appears in the early stage, followed by a defect in insulin secretion, leading to severe hyperglycemia [48], which might be too severe for the treatment of exercise alone to reduce blood glucose levels. The results of [49] are also similar to ours. Furthermore, the maximum exercise capacity of *db/db* mice is much less than that of normal mice [50], which might also explain this.

We used scRNA-seq to investigate the responses of pancreatic cells to exercise, to improve the understanding of the underlying mechanism of exercise-induced alleviation of pancreatic dysfunction in T2DM mice.

Our analysis revealed that among the 12 cell types identified, *db/db* mice exhibited a greater abundance of ECs than *db/m* mice. Notably, exercise was observed to effectively reduce the number of ECs in *db/db* mice. The EC-lined blood vascular system is composed of various vascular beds, including capillaries, arteries, and veins, which play a critical role in the circulation of blood. Additionally, lymphatic vessels, which are also lined with ECs, are involved in the drainage of fluid from the extravascular space [51].

ECs within various tissues display distinct characteristics, likely to adapt to the unique physiological demands of each tissue [52]. Meta et al. [43] reported that ECs from different tissues activated distinct sets of metabolic genes. Nevertheless, the specific EC subcluster in the pancreas that is involved in T2DM has not yet been identified. Through the analysis of the differential expression of established markers for various EC subtypes, we annotated the EC subclusters and inferred their potential functions. This analysis revealed the presence of two previously unknown EC subtypes.

The results show a higher percentage of capillary ECs in the *db/db* group than in the *db/m* group, and exercise reversed the increase in the percentage of capillary ECs in the *db/db* mice. However, the trend observed in the unknown EC 1 was opposite to that in capillary cells, suggesting that both capillary ECs and unknown EC 1 may play important roles in the positive effect of exercise on pancreatic injury in *db/db* mice.

However, the function of these two EC subtypes in the pancreas remains unclear.

The results of the GO enrichment analysis showed that the investigated target marker genes of capillary ECs could be involved in biological processes such as vascular development, regulation of vascular development, regulation of EC proliferation, response to interferon-beta, regulation of supramolecular fiber organization, platelet activation, signaling and aggregation, blood vessel EC migration, positive regulation of programmed cell death, regulation of apoptotic pathways, and regulation of inflammatory responses.

Angiogenesis is stimulated by hypoxic and inflammatory environments and is the process where new blood vessels grow from the existing vasculature. The formation of new blood vessels, a process known as angiogenesis, is stimulated by hypoxic and inflammatory environments, involving several fundamental steps: degradation of the basement membrane of capillaries, migration and proliferation of ECs, formation of lumens, fusion and pruning of ECs, and the recruitment of pericytes to provide structural support [53, 54].

Vessel-resident vascular endothelial stem cells (VESCs) have been reported to be responsible for angiogenesis and are endothelial colony-forming cells (ECFCs) [55–58]. Single VESCs can produce thousands of ECs in vitro and, following transplantation, form functional blood vessels in three dimensions in vivo, indicating the stem cell characteristics of VESCs. Wakabayashi et al. [59] discovered that BM stromal antigen-1/Bst1/CD157 is a marker of hierarchically proliferating VESCs in mouse blood vessels.

Our results showed that Bst1 (Table 3) was expressed only in capillary and venous ECs, which is indicative of the VESC properties of capillary ECs. The augmented count of ECs in capillaries of *db/db* mice suggests that vascular endothelial stem cells within these capillaries are capable of proliferating and acting as the initial cells that initiate angiogenesis. However, we recognize that this is not conclusive evidence, and the claim remains indirect. To confirm VESC involvement, we suggest lineage tracing to track cell differentiation and proliferation and functional assays like colony-forming and transplantation assays to test stem cell–like functions. In future research, we will use these methods to better understand VESCs in capillary ECs and their role in exercise-mediated pancreatic protection in *db/db* mice. This will help clarify the underlying mechanisms and could benefit T2DM-related pancreatic complication treatment.

Furthermore, the GO enrichment analysis revealed the following signaling pathways related to the top marker genes in unknown EC 1: those leading to response to injury and regulation of angiogenesis, positive regulation of cell motility, tubal morphogenesis, homeostasis, cell junction organization, circulatory system processes, extracellular matrix organization, leukocyte cell–cell adhesion, and SRP-dependent cotranslational protein targeting to the membrane.

Our results showed that there was an increase in the percentage of ECs in diabetes, which might have been a compensatory response, as hyperglycemia drives the proliferation of ECs through proangiogenic factors [60, 61], attempting to relieve *β* cell hypoxia by increasing pancreatic islet blood flow. However, the abnormal proliferation of ECs may lead to pathological angiogenesis, which plays a major role in many diabetic tissues, including the pancreas and retina, ultimately inducing inflammation [62–64]. Xiong et al. also demonstrated that obesity induced abnormal pancreatic islet EC proliferation, which altered vessel structure and permeability, resulting in permanent islet vascular dysfunction [65]. It is also reported that endothelial progenitor cells from diabetic mice can regenerate islet ECs but fail to promote *β* cell regeneration, unlike those from normal mice [66], indicating functional impairment despite increased EC numbers in our study, as the crosstalk between ECs and *β* cells—mediated by cytokines (e.g., VEGF, insulin, CTGF, and HGF) that mutually regulate proliferation, hormone secretion, and structural and functional maintenance—might be disrupted in diabetes [64]. Emerging evidence indicates that while islet ECs normally support *β* cell function, they paradoxically exacerbate *β* cell dysfunction and reduce survival under diabetic conditions [64]. At the same time, despite the increase in the number of ECs, the ROS produced by ECs under diabetic states due to its decline in the bioavailability of NO [34] further exacerbates the oxidative damage to *β* cells [35]. Fortunately, it is demonstrated that exercise may reduce oxidative stress by upregulating antioxidant enzymes to protect *β* cells [67]. Evidence also shows that exercise improves endothelial function by increasing NO bioavailability and reducing oxidative stress [38]. Our results showed that exercise, which may improve EC function, restored the number of ECs to a normal level in diabetic mice, resulting in the reduction of ROS production and inflammation induced by the abnormal proliferation of ECs and inhibition of pathological angiogenesis, ultimately leading to *β* cell protection and vascular normalization.

Given that capillary ECs are involved in EC proliferation, while unknown EC 1 are associated with the response to injury and the regulation of angiogenesis, and their expressions in the three groups are diametrically opposed, we have reason to hypothesize the following. In the diabetic state, the number of pancreatic capillary ECs increases, which promotes compensatory proliferation of ECs, leading to pathological angiogenesis and oxidative damage to *β* cells. However, exercise can reverse this phenomenon and promote vascular normalization and protection of *β* cells.

Meanwhile, diabetes reduces the ability of unknown EC 1 to respond to injury. Intriguingly, exercise has the potential to restore this ability. Understanding these relationships may provide new insights into the mechanisms underlying vascular changes in diabetes and the beneficial effects of exercise on pancreas health.

Study limitations: due to the limited number of pancreatic ECs in mice, our next step will be to specifically collect pancreatic ECs from mice using flow cytometry sorting for subsequent analysis.

## 5. Conclusion

In conclusion, our study revealed that diabetes induces an increase in EC percentage as a compensatory response to hyperglycemia, aiming to improve pancreatic injuries. However, this adaptive process may lead to pathological angiogenesis and exacerbated oxidative damage to *β* cells due to reduced NO bioavailability. Conversely, exercise emerged as a key regulator: it may normalize EC numbers in diabetic mice, suppress ROS production, and inhibit pathological angiogenesis. These effects collectively alleviated pancreatic injuries in diabetic mice. Our findings underscore the critical role of ECs in the interplay between diabetes and exercise, suggesting that exercise may offer a multifaceted approach to counteract diabetic pancreatic dysfunction.

These results have significant clinical implications. Future research could explore whether exercise-induced modulation of ECs can be translated into clinical interventions, such as exercise-based therapies or drugs that mimic exercise-mediated EC regulation. Understanding these mechanisms may open new avenues for preventing or treating T2DM-associated vascular and pancreatic damage, ultimately improving patient outcomes.

## Figures and Tables

**Table 1:** Cell type identification results of pancreas cells.

**Cell types**	***db/m***	**%**	***db/db***	**%**	***db/db*-Ex**	**%**
Total	8121	100	9611	100	13,673	100
Acinar cells	6221	76.6	7152	74.41	11,859	86.74
Ductal cells	692	8.53	926	9.63	692	5.06
Fibroblast cells	289	3.55	489	5.09	343	2.51
Macrophage cells	297	3.66	230	2.39	218	1.59
Endothelial cells	143	1.76	586	6.09	390	2.85
B cells	208	2.56	4	0.04	8	0.06
Stellate cells	41	0.5	97	1.01	80	0.59
T cells	176	2.17	16	0.17	21	0.15
Erythroid cells	34	0.42	25	0.26	33	0.24
Endocrine cells	6	0.07	63	0.66	16	0.12
Granulocyte cells	14	0.17	23	0.24	13	0.1

*Note:* The table exhibits cell type identification results of pancreas cells and fraction of cells in each population relative to the total number of pancreatic cells.

**Table 2:** Cell type identification results of pancreatic endothelial cells.

**Cells**	***db/m***	**%**	***db/db***	**%**	***db/db*-Ex**	**%**
Capillary ECs	5	3.5	290	49.49	94	24.1
Unknown EC 1	124	86.71	127	21.68	150	38.46
Unknown EC 2	4	2.8	83	14.16	47	12.05
Arterial ECs	8	5.59	69	11.77	30	7.69
Venous ECs	2	1.4	17	2.9	69	17.69

*Note:* The table exhibits cell type identification results of pancreas endothelial cells and fraction of cells in each population relative to the total number of pancreatic endothelial cells.

**Table 3:** The expression levels of Bst1.

**Cluster**	**Arterial_ECs**	**Capillary_ECs**	**Unknown_ECs_1**	**Unknown_ECs_2**	**Venous_ECs**
Bst1	0	0.05	0	0	0.47

*Note:* The table exhibits the expression levels of Bst1 among five endothelial subtype cells. Data is from analysis of the FindAllMarkers function in the Seurat package.

## Data Availability

The data that support the findings of this study for reviewer's access are available in NCBI SRA Bioproject at https://dataview.ncbi.nlm.nih.gov/object/PRJNA1149948?reviewer=2bjqhnmqrhqdsveaheufb692m6.

## Supporting Information

Supporting Information 1 Material S1. The tables and figure display quality control metrics. Table S1. The table displays the comparison statistics of single-cell sequencing samples based on Cell Ranger, which helps to evaluate the quality of sequencing data and the comparison effect and provides an important basis for subsequent analysis. Table S2. The table exhibits the sequencing statistics of single-cell sequencing samples based onc. Table S3. The table displays the statistical information of the filtered sample cells based on the advanced analysis of single-cell data via Seurat. Figure S1. Distribution of basic cell information of each sample before and after cell filtering. (A) Distribution of basic cell information of each sample before filtering. (B) Distribution of basic cell information of each sample after filtering. For each group of images, the left-hand graph shows the distribution of the number of genes detected in single cells of each sample (*y*-axis); the middle graph shows the distribution of the total number of unique molecular identifiers (UMIs) detected in single cells of each sample (*y*-axis); the right-hand graph shows the distribution of the percentage of mitochondrial gene expression in single cells of each sample (*y*-axis). (Note: Poor-quality cells usually have abnormally high mitochondrial gene expression, and the mitochondrial gene expression level can be used as a reference index to identify low-quality cells in the sample.)

Supporting Information 2 Material S2. The table displays the top 10 differentially expressed genes (DEGs) for each cell type in the pancreas.

Supporting Information 3 Material S3. The table displays the top 10 DEGs of the subcell types of pancreatic ECs.
