# Supplementary material for: Single-Cell RNA Sequencing Shows Exercise Protects db/db Mouse Pancreatic Injuries by Regulating Endothelial Cell Homeostasis
Source: J Diabetes Res. 2025 Sep 22;2025:9676094. doi: 10.1155/jdr/9676094 (PMC12479157; doi:10.1155/jdr/9676094)
Supplement: Supporting Information 1 — Material S1. The tables and figure display quality control metrics. Table S1. The table displays the comparison statistics of single-cell sequencing samples based on Cell Ranger, which helps to evaluate the quality of sequencing data and the comparison effect and provides an important basis for subsequent analysis. Table S2. The table exhibits the sequencing statistics of single-cell sequencing samples based onc. Table S3. The table displays the statistical information of the filtered sample cells based on the advanced analysis of single-cell data via Seurat. Figure S1. Distribution of basic cell information of each sample before and after cell filtering. (A) Distribution of basic cell information of each sample before filtering. (B) Distribution of basic cell information of each sample after filtering. For each group of images, the left-hand graph shows the distribution of the number of genes detected in single cells of each sample (y-axis); the middle graph shows the distribution of the total number of unique molecular identifiers (UMIs) detected in single cells of each sample (y-axis); the right-hand graph shows the distribution of the percentage of mitochondrial gene expression in single cells of each sample (y-axis). (Note: Poor-quality cells usually have abnormally high mitochondrial gene expression, and the mitochondrial gene expression level can be used as a reference index to identify low-quality cells in the sample.) [file 9676094.f1.docx]

| Supplemental. Table. 1 The comparison statistics of single-cell sequencing samples | | | |
| --- | --- | --- | --- |
| Sample | *db/m* | *db/db* | *db/db*-Ex |
| Estimated Number of Cells | 9,958 | 12,743 | 18,433 |
| Mean Reads per Cell | 31,138 | 25,178 | 21,231 |
| Median Genes per Cell | 413 | 457 | 254 |
| Reads Mapped to Genome | 95.6% | 89.5% | 87.5% |
| Reads Mapped Confidently to Genome | 75.5% | 76.0% | 67.4% |
| Reads Mapped Confidently to Intergenic Regions | 0.5% | 0.8% | 0.9% |
| Reads Mapped Confidently to Intronic Regions | 1.1% | 1.7% | 2.0% |
| Reads Mapped Confidently to Exonic Regions | 73.8% | 73.5% | 64.5% |
| Reads Mapped Confidently to Transcriptome | 68.6% | 69.0% | 60.1% |
| Reads Mapped Antisense to Gene | 0.4% | 0.4% | 0.4% |
| Fraction Reads in Cells | 36.3% | 60.4% | 48.5% |
| Total Genes Detected | 21,869 | 23,325 | 22,671 |

**Supplemental. Table. 1** The table displays the comparison statistics of single-cell sequencing samples based on CellRanger, which helps to evaluate the quality of sequencing data and the comparison effect, and providing an important basis for subsequent analysis.

| Supplemental. Table. 2 The sequencing statistics of single-cell sequencing samples | | | |
| --- | --- | --- | --- |
| Sample | *db/m* | *db/db* | *db/db*-Ex |
| Number of Reads | 310,069,273 | 320,847,140 | 391,343,472 |
| Valid Barcodes | 98.3% | 98.9% | 98.9% |
| Sequencing Saturation | 30.0% | 40.1% | 61.3% |
| Q30 Bases in Barcode | 95.7% | 95.7% | 95.9% |
| Q30 Bases in RNA Read | 95.0% | 94.2% | 94.2% |
| Q30 Bases in UMI | 95.1% | 95.1% | 95.2% |

**Supplemental. Table. 2** The table exhibits display the sequencing statistics of single-cell sequencing samples based onc.

| Supplemental. Table. 3 The statistical information of the filtered sample cells | | | |
| --- | --- | --- | --- |
| Sample | *db/m* | *db/db* | *db/db*-Ex |
| before_filter_cell_num | 18433 | 12743 | 9958 |
| after_filter_cell_num | 13673 | 9611 | 8121 |
| precent | 74.18% | 75.42% | 81.55% |

**Supplemental. Table. 3** The table displays the statistical information of the filtered sample cells based on the advanced analysis of single-cell data via Seurat.


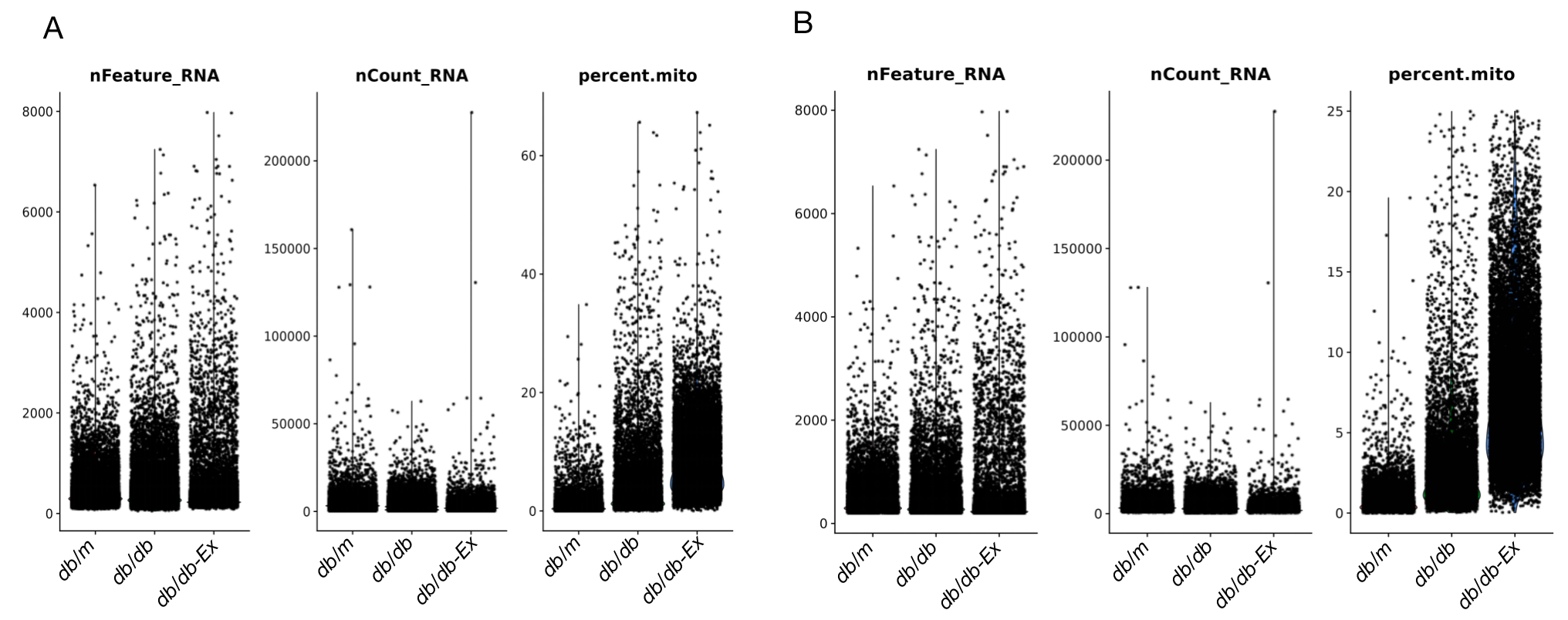


**Supplemental. figure. 1** Distribution of Basic Cell Information of Each Sample Before and After Cell Filtering

A: Distribution of basic cell information of each sample before filtering; B Distribution of basic cell information of each sample after filtering.

For each group of images, the left-hand graph shows: the distribution of the number of genes detected in single cells of each sample (Y-axis); the middle graph shows: the distribution of the total number of unique molecular identifiers (UMIs) detected in single cells of each sample (Y-axis); the right-hand graph shows: the distribution of the percentage of mitochondrial gene expression in single cells of each sample (Y-axis). (Note: Poor-quality cells usually have abnormally high mitochondrial gene expression, and the mitochondrial gene expression level can be used as a reference index to identify low-quality cells in the sample.)
